# Telemetric Interventions Offer New Opportunities for Managing Type 1 Diabetes Mellitus: Systematic Meta-review

**DOI:** 10.2196/20270

**Published:** 2021-03-16

**Authors:** Claudia Eberle, Stefanie Stichling

**Affiliations:** 1 Medicine with Specialization in Internal Medicine and General Medicine Hochschule Fulda - University of Applied Sciences Fulda Germany

**Keywords:** type 1 diabetes, telemetry, telemedicine, telemonitoring, digital health, eHealth, diabetes management, systematic meta-review

## Abstract

**Background:**

The prevalence of diabetes mellitus (DM) is increasing rapidly worldwide. Simultaneously, technological advances are offering new opportunities for better management of type 1 diabetes mellitus (T1DM). Telemetry, the remote acquisition of patient data via a telecommunication system, is a promising field of application in eHealth and is rapidly gaining importance.

**Objective:**

The aim of this study was to summarize the current evidences available on the effectiveness of telemetric approaches in T1DM management. This systematic meta-review examined different types of interventions of the technologies used in communication between health care professionals and patients as well as the key outcomes.

**Methods:**

We performed a systematic search in Web of Science Core Collection, EMBASE, Cochrane Library, MEDLINE via PubMed, and CINAHL databases in April 2020 with regard to the effectiveness of telemetric interventions for T1DM. We classified the interventions into 4 categories according to the technology used: (1) real-time video communication, (2) real-time audio communication, (3) asynchronous communication, and (4) combined forms of communication (real-time and asynchronous). We considered various study designs such as systematic reviews, clinical trials, meta-analyses, and randomized controlled trials and focused on the key outcomes. Additionally, a funnel plot based on hemoglobin A_1c_ (HbA_1c_) values and different quality assessments were performed.

**Results:**

We identified 17 (6 high quality and 9 moderate quality) eligible publications: randomized controlled trials (n=9), systematic reviews and meta-analyses (n=5), cohort studies (n=2), and qualitative publications (n=1). Of 12 studies, 8 (67%) indicated a (significant or nonsignificant) reduction in HbA_1c_ levels; 65% (11/17) of the studies reported overall (mildly) positive effects of telemetric interventions by addressing all the measured outcomes. Asynchronous interventions were the most successful for patients diagnosed with T1DM, but no technology was clearly superior. However, there were many nonsignificant results and not sustained effects, and in some studies, the control group benefited from telemetric support or increased frequency of contacts.

**Conclusions:**

Based on the currently available literature, this systematic meta-review shows that telemetric interventions cause significant reduction in HbA_1c_ levels and result in overall positive effects in T1DM management. However, more specified effects of telemetric approaches in T1DM management should be analyzed in detail in larger cohorts.

## Introduction

The historical origins of digital health date back to the 1970s, when telematics, the science of telecommunications and informatics, emerged [1]. Telemedicine developed as a technology-supported physician-patient relationship in the 1970s/80s as a subarea of telematics. In the 1990s, the emergence of the internet resulted in new communication channels and the development of eHealth [1]. Mobile health, which was developed as a subarea of eHealth in 2010, is referred by the World Health Organization as “medical and public health practice supported by mobile devices such as mobile phones, patient monitoring devices, personal digital assistants, and other wireless devices” [2]. Nowadays, digital health defines the intersection of digital transformations with health, life, and communities [3].

Telemedicine is a digital field of application and part of eHealth and digitalization in the health care sector [4]. The exchange between different user groups (eg, physician, patient, service provider) takes place in these apps [5]. When integrating users in the area of eHealth, and thus in telemedicine, a distinction is made between different forms of communication structures. This review focuses on the communication structure of “physician to patient,” which defines the communication between physicians (or health care professionals) and patients [5]. Telemetry has the advantage that no physical presence is necessary [6]. Telemetry is characterized by the American Telemedicine Association as “remote acquisition, recording, and transmission of patient data via a telecommunications system to a health care professional for analysis and decision making” [6]. In telemetric interventions, patients upload data (eg, dietary habits and glucose levels) and health care professionals review these data and offer feedback (eg, regarding medication and lifestyle) [6,7]. In this regard, telementoring describes the use of telecommunications (eg, audio or video) and electronic information processing technologies to provide those customized instructions [6].

This systematic meta-review focuses on telemetry by using the example of patients diagnosed with type 1 diabetes mellitus (T1DM). DM is one of the most prevalent chronic diseases worldwide [8]. Globally, approximately 463 million adults (age range 20-79 years) are diagnosed with DM [8]. T1DM accounts for 5%-10% of all DM forms and can arise at any age; however, it is frequently reported in kids and young adults [8]. The prevalence of T1DM has been increasing in the past decades. Globally, about 1.1 million children and adolescents (age range 0-19 years) are diagnosed with T1DM [8]. From a pathophysiological and a clinical view, T1DM is a very complex disease, which is dependent on beta-cell demolition by the T cells of the immune system, resulting in the total lack of insulin [9]. Comorbidities such as microvascular (eg, nephropathy, retinopathy, and neuropathy) and macrovascular (eg, cardiovascular disease, stroke) complications are closely and frequently related to DM [9]. Optimal glycemic control is *the* therapy goal to reduce and prevent such diabetic complications and comorbidities. Intensive therapeutic measures address the delay of onset of diabetic complications as well as comorbidities in T1DM [10]. Therefore, technological advances in diabetes therapy may provide powerful novel solutions for a better and more closed-meshed disease management [11]. Several studies have examined the capability of telemetry in the treatment of DM [12-14]. The use of technological apps may be an attractive option for T1DM management. Previous studies have shown feasibility and satisfaction by using telemedicine [13,14]. However, the evidence for the impact of telemetric interventions in the context of diabetes therapy and the potential of these interventions should be examined further. Therefore, this systematic meta-review intended to assess the current evidence for the effectiveness of telemetric interventions in the management of T1DM. Not only randomized controlled trials (RCTs), as it is often the case in the literature, but also various study designs, including clinical trials, systematic reviews, and meta-analyses, were considered.

## Methods

### Search Strategy

We performed a systematic search in Web of Science Core Collection, EMBASE, Cochrane Library, MEDLINE via PubMed, and CINAHL databases in April 2020. The systematic meta-review was carried out based on the Preferred Reporting Items for Systematic Reviews and Meta-Analyses (PRISMA) guidelines [15]. Peer-reviewed full-text publications assessing the effectiveness of telemetric interventions in patients with T1DM, published from 2008 to April 2020, were included. We selected keywords from the medical subject headings and EMBASE subject headings databases and used title/abstract terms. The following Boolean logic was applied: (Diabetes Mellitus) AND (Telemetry OR Telemonitoring OR Telemedicine). No restrictions for geographical locations were placed. Initially, we carried out an extensive literature search with a strategy that covered different types of DM (T1DM, type 2 DM [T2DM], and gestational DM). During the process, T1DM studies were selected for this systematic meta-review. We additionally carried out manual researches of the references of the included examinations to recognize other reasonable publications. All search terms for the individual databases are provided in Multimedia Appendix 1.

### Inclusion Criteria

We included publications written in English and German with the target group patients diagnosed with T1DM. These publications addressed interventions in the field of telemetry, telemedicine, and telemonitoring for their diabetes therapy. The intervention involved direct interaction between the patients and health care professionals, that is, feedback from health care professionals based on the transmitted patient data. We included the following study designs: systematic reviews, meta-analyses, clinical trials, and RCTs.

### Exclusion Criteria

**Figure 1:**
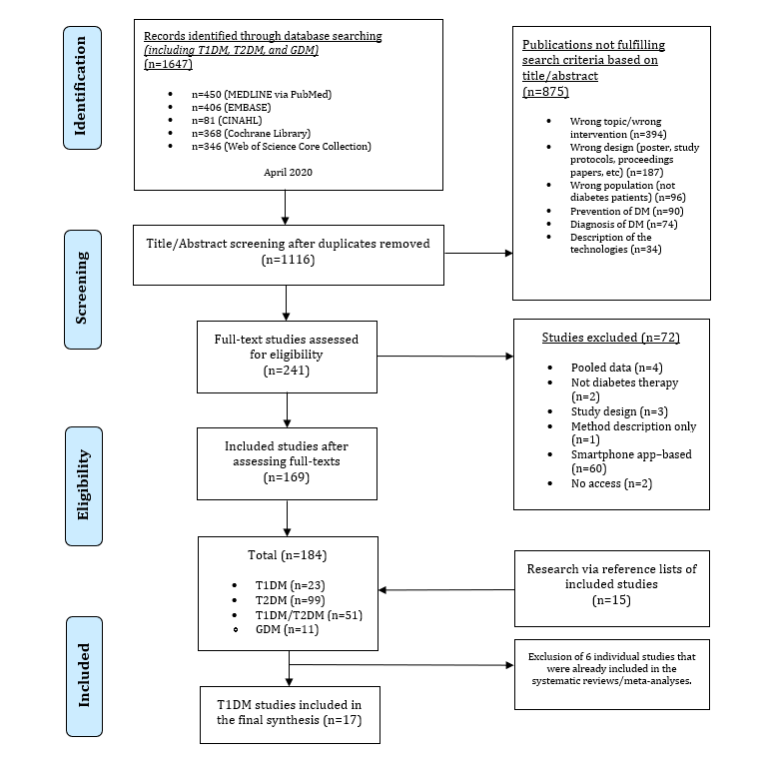
PRISMA flowchart of the procedure for the search and selection of suitable publications (adapted from Moher et al [15]). GDM: gestational diabetes mellitus; T1DM: type 1 diabetes mellitus, T2DM: type 2 diabetes mellitus.

Since this systematic meta-review focused on T1DM, we excluded participants diagnosed with other forms of DM (such as T2DM, gestational DM, and other types of diabetes) as well as mixed collectives, meaning that studies included not only patients with T1DM but also people diagnosed with other types of DM. Moreover, we excluded individual studies that were already included in the identified systematic reviews and meta-analyses; therefore, no data from systematic reviews/meta-analyses and individual studies are pooled, leading to a possible bias. Abstracts, posters, comments, letters, study protocols, notes, and proceedings papers were excluded. In addition, publications that focused on the description of the technology were rejected. Telemetry is a wide term and may cover different technologies. Since the way of communication between patients and health care professionals is different compared to that in telemetric interventions, we analyzed interventions with mobile apps in other studies separately. We also eliminated studies providing only pooled data (ie, with patients of other diseases and with digital apps other than telemetry). Furthermore, duplicates and studies that addressed prevention or diagnosis of DM were rejected. The literature search is documented in the PRISMA flowchart (Figure 1). As Figure 1 shows, we selected T1DM studies from our extensive literature search.

### Data Extraction

We extracted the year of publication, study designs, durations, intervention and control groups, outcome measures, sample sizes, country, statistical significances, and conclusions. Intervention and control group data included the technologies used, feedback methods, the frequency of contact, and data transmission. The significance involved the comparison of the intervention group with the control group (intergroup) and the comparison within the intervention group, that is, from the baseline to the end of the study (intragroup), depending on what was reported. In relation to the systematic reviews and meta-analyses, the *overall* effects were extracted (overall positive effect, no effect, or inconclusive results). The quality of life (QoL) was divided into diabetes-related quality of life (DRQoL) as well as health-related quality of life (HRQoL).

### Data Synthesis and Analysis

A qualitative analysis was conducted. The selected studies differed regarding sample, design, and measures. A proper meta-analysis was therefore not possible. For analysis, the studies were classified into different categories based on a scheme that we developed. First, the publications were systematized into 4 categories according to the technologies used to communicate between the health care professionals and the patients (Textbox 1).

**Categories for the classification of the different intervention types.**

**Different intervention types**

- Real-time communication video: Synchronous face-to-face communication by videoconferencing and videoconsulting.
- Real-time communication audio: Synchronous communication by telephone calls (telephone coaching and counselling).
- Asynchronous communication: Asynchronous communication by email, SMS text messaging, internet/web-based platforms, server, home gateway, or post.
- Combined forms of communication: The intervention involves real-time and asynchronous communication.

**Figure 2:**
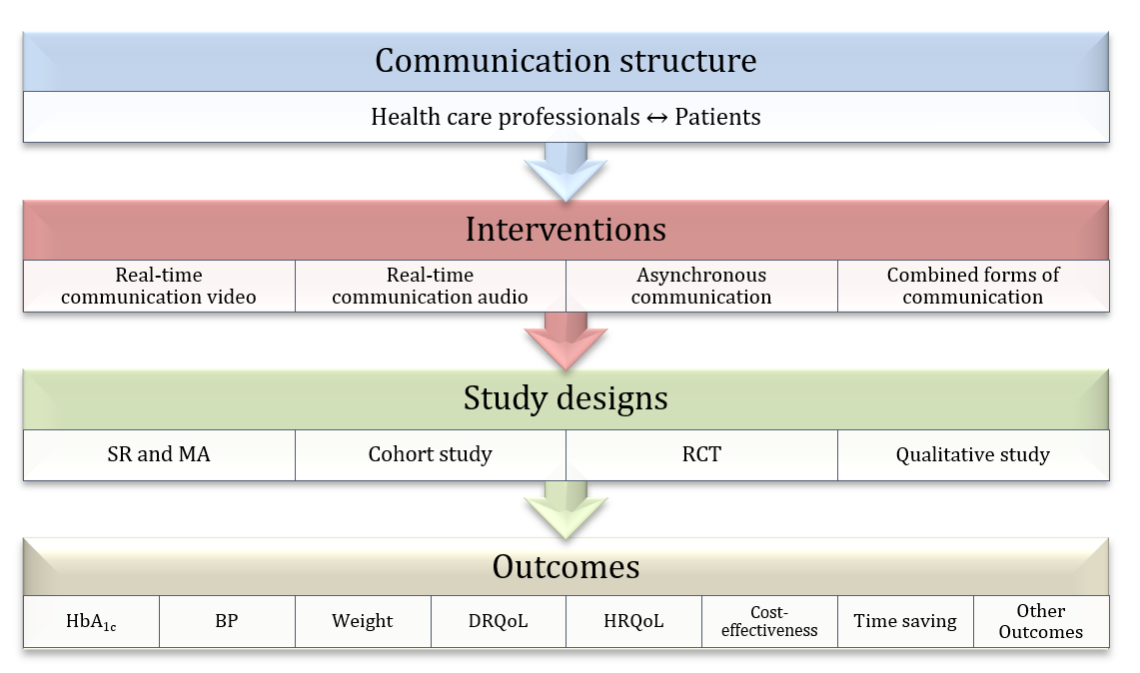
Scheme for structuring the included studies. BP: blood pressure; HbA_1c_: hemoglobin A_1c_; DRQoL: diabetes-related quality of life; HRQoL: health-related quality of life; MA: meta-analysis; SR: systematic review; RCT: randomized controlled trial.

Due to the heterogeneity, systematic reviews and meta-analyses were not assigned to these categories. Second, the studies were differentiated according to their designs. Third, these were structured based on key outcomes: hemoglobin A_1c_ (HbA_1c_), body weight, blood pressure, QoL, cost-effectiveness, and time saved (Figure 2).

### Assessment of Risk of Bias

A quality assessment of the studies was conducted to determine the risk of bias. Since we included different study designs, we applied 3 different quality appraisal tools. First, we applied A MeaSurement Tool to Assess systematic Reviews (AMSTAR 2), a validated and widely used tool for the evaluation of systematic reviews and meta-analyses. AMSTAR 2 rates the study quality as high, moderate, low, or critically low. Second, we used Effective Public Health Practice Project (EPHPP), a validated instrument that addresses studies on health-related topics. Since this tool is suitable for quantitative intervention studies, we used it for RCTs and cohort studies. EPHPP consists of the following components: selection bias, study design, confounders, blinding, data collection methods, and withdrawals and drop-outs. The instrument rates the study quality as strong, moderate, or weak. Third, we applied the validated National Institute for Health and Care Excellence (NICE) quality appraisal checklist for qualitative studies. The NICE checklist includes the following components: theoretical approach, study design, data collection, trustworthiness, analysis, and ethics. This tool rates the study quality as ++ (high), + (moderate), or – (low). In addition, the publication bias was assessed visually as a funnel plot by using HbA_1c_ values. The studies were extremely heterogeneous. Without systematic reviews, meta-analyses, and cohort studies (ie, without control group) and excluding a study that compared 2 telemetric applications, we generated a funnel plot based on 6 RCTs. Intervention effect was expressed as the mean difference using HbA_1c_ values at the end of the study.

## Results

### Study Characteristics

The database search resulted in 1647 records. After removing duplicates, 1116 publications were screened for eligibility. We excluded 875 of these records based on titles/abstracts for the reasons given in Figure 1. After reviewing 241 full-text publications and an additional research of reference lists, a total of 189 studies were identified (T1DM, n=23; T2DM, n=99; gestational DM, n=11; and both T1DM/T2DM, n=51). We excluded 6 individual studies [16-21] that were already involved in the systematic reviews/meta-analyses. Finally, 17 publications were included in this synthesis. Multimedia Appendix 2 provides a detailed summary of each publication selected for inclusion in this systematic meta-review, including all measured outcomes. Table 1 shows the features of the included studies. Most studies (with exception of systematic reviews and meta-analyses due to their heterogeneity) were performed in Europe (n=6), followed by in the United States (n=3), Asia (n=1), and Russia (n=1), along with not specified (n=1). We categorized the studies by the type of intervention: real-time communication via video (n=3), asynchronous communication (n=4), and combined forms of communication (n=4). One qualitative study did not explain the intervention in detail. No real-time audio interventions were identified. Most studies were RCTs (n=9), systematic reviews and meta-analyses (n=5), as well as cohort studies (n=2), and qualitative publications (n=1). A presentation of all the intervention effects (significant and nonsignificant) on the key outcomes is provided in Multimedia Appendix 3. Two systematic reviews and meta-analyses were assessed as high-quality studies, whereas 2 were rated as moderate and 1 as critically low quality. Of the real-time video interventions, 3 were high-quality studies. Furthermore, 4 asynchronous interventions were rated as moderate quality. Of the combined interventions, 1 was rated as high, 2 as moderate, and 1 as weak-quality study. In addition, the qualitative publication was of moderate quality. The detailed quality appraisals are presented in Multimedia Appendix 4.

**Table 1:** Baseline characteristics of all the included publications.

Characteristics of the publications				Values, n (%)
**Study design (n=17)**				
	Systematic reviews and meta-analyses (total)			5 (29)
	Randomized controlled trial (total)^a^			9 (53)
	Cohort (total)^b^			2 (12)
	Qualitative (total)			1 (6)
**Year of publication (n=17)**				
	2008-2011			2 (12)
	2012-2014			4 (24)
	2015-2017			5 (29)
	2018-2020			6 (35)
**Excluding systematic reviews and meta-analyses (n=12)**				
	**Location**			
		United States	3 (25)	
		Europe	6 (50)	
		Asia	1 (8)	
		Russia	1 (8)	
		Not specified	1 (8)	
	**Intervention type**			
		Real-time video	3 (25)	
		Asynchronous	4 (33)	
		Combined forms	4 (33)	
		Not specified	1 (8)	

^a^This included 1 pilot randomized controlled trial.

^b^This included 1 pilot cohort study.

HRQoL and DRQoL were evaluated using very different methods. Validated instruments were used to measure these outcomes, for example, 36-item Short Form Health Survey, Diabetes Quality of Life questionnaire, PedsQLTM 3.0 Diabetes Module questionnaire, 12-item Short Form Health Survey, and European Quality of Life survey. There were also specially designed questionnaires.

### Effectiveness of Telemetry: Key Outcomes

Of 17 studies, 11 (65%) reported overall (mildly) positive effects of the telemetric interventions in relation to all measured outcomes (Multimedia Appendix 2). Table 2 presents the significant effects (intragroup and intergroup) on the key outcomes. Of 12 studies, 8 (67%) indicated a (significant or nonsignificant) reduction (intragroup or intergroup) in HbA_1c_ levels in the intervention group. Descriptive examination of the funnel plot by using HbA_1c_ values based on 6 RCTs indicated a mild form of asymmetry (Multimedia Appendix 5).

**Table 2:** Impact of the interventions on selected outcomes (intragroup and intergroup) (n=17).^a^

Outcomes/ interventions	Hemoglobin A_1c_	Blood pressure	Body weight	Diabetes-related quality of life	Health-related quality of life	Costs	Time saved	Others or not significant
Systematic review and meta-analysis	3	—^b^	—	1	—	—	—	—
Real-time video^c^	—	—	—	—	—	—	—	✓
Asynchronous	1	—	—	—	—	—	—	—
Combined	1	—	—	1	—	—	—	—
Not specified^c^	—	—	—	—	—	—	—	✓

^a^All studies that reported significant intervention effects are mentioned in this table, including those effects that were not sustainable. This table does not include studies reporting nonsignificant intervention effects. The values in the tables indicate the number of studies that examined the outcome and these studies showed improvement in that particular outcome.

^b^Not available.

^c^Studies in this category did not examine any of the listed outcomes nor report any significant effects.

### Systematic Reviews and Meta-Analyses

#### HbA_1c_ Levels (n=5)

All 5 systematic reviews and meta-analyses analyzed HbA_1c_ levels as the targeted outcome. Three studies (60%) reported overall positive effects in terms of reducing HbA_1c_ levels significantly. Lee et al [12] (high-quality study) described a mean reduction of 0.18% (95% CI 0.04-0.33, *P*=.01). Peterson [22] (critically low-quality study) outlined that 12 studies showed a decline in HbA_1c_ levels in their intervention groups. However, Viana et al [23] (moderate-quality study) and Shulman et al [24] (high-quality study) found no significant decrease in HbA_1c_ levels following telemedical interventions (mean deviation –0.124%, 95% CI, –0.268 to 0.020; *P*=.09 [25] and mean deviation –0.12, 95% CI, –0.35 to 0.11; *P*>.05 [24], respectively).

#### Blood Pressure and Body Weight (n=1)

Lee et al [12] (high-quality study) observed no benefits through telemedicine on either blood pressure or body weight.

#### DRQoL (n=3) and HRQoL (n=1)

Three studies examined the DRQoL. Two high-quality studies (67%) found no effects [12,24] and a moderate-quality review [26] that only included 1 suitable study found a significant improvement in DRQoL. In addition, 1 review observed no benefits on generic HRQoL [12].

#### Cost-Effectiveness (n=1)

One high-quality study described that the limited data available on the costs of telemedicine suggested no differences between the groups [24]. One of the included studies of this review reported that the intervention group omitted the 3-month visit, which saved US $142 [24].

### Asynchronous Interventions

#### HbA_1c_ Levels (n=3)

A cohort study (moderate quality) reported significantly reduced mean HbA_1c_ levels at the end of the assessment phase (*P*=.01) [27]. However, another 2 moderate-quality RCTs found no significant differences HbA_1c_ values between groups (*P*=.84 [28] and *P*=.49 [29]). One of these studies [28] examined telemedicine in addition to conventional care in the intervention group.

#### HRQoL (n=1)

One moderate-quality RCT observed that changes in HRQoL between the first visit and the final visit did not differ between the groups [30].

### Combined Interventions

#### HbA_1c_ Levels (n=4)

All 4 RCTs considered the outcome HbA_1c_. Only 1 study (moderate quality) showed significant improvements in the HbA_1c_ levels in the patients undergoing interventions (8.7% to 7.7%) compared to the controls (8.7% to 8.4%, *P*<.05) [31]. Gandrud et al (weak-quality study) [32] and Yaron et al [25] (high-quality study) reported positive but no significant differences in the effects on HbA_1c_ levels between the telemedicine and usual care groups. In addition, 1 moderate-quality publication mentioned no improvement in HbA_1c_ levels, with no statistically significant difference (*P*=.56 for control group, *P*=.45 for telemetry group, and *P*=.60 between groups) [33].

#### DRQoL (n=2)

According to an RCT (weak-quality study), a number of QoL indicators increased significantly due to telemetry compared to that in the control group (*P*<.05) [31]. However, another moderate-quality RCT showed no significant increase in QoL by 6.5 points and 1.3 points for intervention group and control group (*P*=.06), respectively [32].

#### Cost-Effectiveness (n=2) and Time Saved (n=1)

Yaron et al (high-quality study) [25] and Bertuzzi et al (moderate-quality study) [33] reported a cost reduction through telemedicine (no significance reported). Direct expenses were 24% lesser in the intervention group, while indirect costs diminished by 22% [25]. One of these studies also mentioned that patients saved time for each visit (mean 115 [SD 86] min) [33].

## Discussion

### Principal Results

This systematic meta-review highlighted the variety of telemetric interventions and technologies used in diabetes care by focusing on T1DM management. Considering all the study designs, asynchronous interventions were found to be the most successful for people with T1DM in improving the key diabetic outcomes, but no technology was clearly superior. However, the results might be inconsistent in terms of the different key outcomes, but fortunately, an improvement in terms of HbA_1c_ values was found. HbA_1c_ was by far the most investigated outcome in these studies. Overall, most systematic reviews and meta-analyses (high and moderate quality) showed a significant reduction in HbA_1c_ values. The other systematic reviews and meta-analyses also indicated positive effects, but they were not statistically significant. The study of Lee et al [12], a high-quality study, achieved a significant and clear reduction of –0.18% (95% CI 0.04-0.33, *P*=.01). Moreover, HbA_1c_ levels were improved significantly in most asynchronous interventions. HbA_1c_ values clearly decreased when combined interventions (asynchronous and real-time communication) were applied, but 1 moderate-quality study showed significant improvements and 3 more (high, moderate, and weak quality) reported positive but not significant effects. Our findings indicated a trend toward better glycemic control for patients with T1DM by means of telemedicine. This result has potential practical implications. The fact that HbA_1c_ levels could be significantly improved in many studies is a promising result in view of the fact that an optimized glycemic control reduces the risk of comorbidities and complications as well as progression of microvascular and macrovascular consequences among patients with T1DM [10]. However, there are only few results for the other outcomes to be able to reach firm inferences. Blood pressure and body weights were examined by 1 meta-analysis. Lee et al (high-quality study) noticed that there are only few studies available revealing no obvious benefits [12]. Aside from that, 2 systematic reviews and meta-analyses (high and moderate quality) outlined no effects in terms of QoL, but a moderate-quality study demonstrated positive tendencies in improving the QoL. Overall, the studies reported that data availability is limited and further investigations are needed. Besides, DRQoL improved significantly in the “real-time video intervention” with weak quality. The moderate-quality asynchronous intervention showed no differences in HRQoL. However, DRQoL also improved obviously in combined interventions, that is, significantly in a weak-quality study and not significantly in a moderate-quality study. In general, there were only few studies on the cost-effectiveness of telemetric interventions. Costs were significantly reduced through “asynchronous interventions,” which was shown by a high-quality study. This high-quality study also demonstrated significant time saving through the asynchronous intervention. With combined interventions, 2 moderate-quality studies also showed clear cost reductions.

In our view, telemetry enables close diabetes management and offers the advantage of overcoming the physical presence. Telemetric technologies allow a higher frequency of contacts between patients and health care professionals. Telemetric interventions also increase, in our view, patient compliance, reliance, and empowerment. The patients implement recommendations for action more successfully in everyday life. They are supervised and managed effectively and more closely and may feel more secure in terms of diabetes therapy. Another systematic review and meta-analysis [12] that recently examined telemetry for the management of clinical outcomes of T1DM also showed that the evidence regarding body weight and blood pressure is clearly limited. In practice, considering the restricted availability of resources, it is important whether the telemetric interventions are cost-effective and time-saving. Therefore, these outcomes are of major importance and should be considered more often in studies in future. Interestingly and surprisingly, fasting blood glucose values seem to be a neglected outcome in these T1DM studies. Since accurate blood sugar measurements are required to reach euglycemic conditions with appropriate insulin doses [9], this outcome is very important.

The systematic reviews and meta-analyses were heterogeneous since telemetry can cover various interventions and technologies and the authors used different definitions of telemedical approaches. Additionally, the variability of the methods used in the studies made it difficult to reach firm conclusions. Studies often suffered from small sample sizes, poor study designs, lack of controls, or no long-term intervention effects. Some studies had samples of patients with poorly controlled diabetes that led to greater intervention effects. Overall, there were not many significant results both for intergroup and intragroup comparisons.

Interestingly, the control group was often not a real or pure control group with usual care. The control group often had an increased frequency of contacts with health care professionals (more than 4 times a year), which led to improved outcomes. In some studies, the control group benefited from telemetric support. Moreover, several studies did not adequately define usual care. The intervention effects might be greater if the telemetric group was compared to a pure control group. Besides, the high number of nonsignificant results is particularly noticeable. This could be related to an often low statistical power. It is also concerning that some studies did not publish *P* values. Furthermore, based on the findings, the long-term effects can be questioned. Some studies found significant positive postintervention effects, but they did not last for a long term. Long follow-up periods are therefore important.

Our review is, as far as we know, the first systematic meta-review on telemedicine in T1DM management. Compared to other papers, this systematic meta-review included different study designs, looked at a variety of outcomes, and carried out a differentiated analysis based on a developed scheme. We also analyzed the findings in detail and differentiated them based on the intergroup or intragroup comparison, significant or not significant effects, and effect sizes. In this way, we were able to contribute to a multifaceted view of the topic.

### Limitations

Some limitations have to be considered when interpreting and using the results. To the best of our knowledge and the elected inclusion and exclusion criteria, we included all suitable studies. Some of the systematic reviews and meta-analyses reported that the poor quality of the included studies was a weakness. Furthermore, numerous definitions of telemetry and telemedicine include different technologies. For the reasons mentioned above, we decided to exclude smartphone app–based interventions, which may be a limitation. Besides, the definition of usual care was insufficient and heterogeneous across the publications. Some studies did not use a control group in the sense of usual care. It is notable that in some studies, the control group had a similar frequency of contacts as the intervention group. In some studies, the control group received telemetric support. These circumstances influence the results achieved and must be considered. Overall, the studies displayed different characteristics and methods, which lead to heterogeneity and can influence the reliability of the results.

### Comparison With Prior Work

In a nutshell, other reviews showed similar inconsistent findings. Lee et al [12] observed no benefits in the interventions with telemedicine focused on blood pressure, body weight, and QoL in 38 RCTs. The overall value of the included interventions was insufficient for glycemic control and other clinical outcomes among patients with T1DM. Viana et al [23] examined telecare interventions to improve patients’ compliance and HbA_1c_ values and found no decrease in HbA_1c_ levels after telecare (*P*=.09). Another systematic review [34] mentioned that 7 of the 14 included publications indicated statistically significant decreases in the observed outcomes, while 79% mentioned success with their telemetric interventions. Baron et al [35] investigated the effectiveness of mobile monitoring technologies for HbA_1c_ levels in 24 studies and found inconsistent evidence for T1DM.

### Conclusions

This systematic meta-review offered a comprehensive summary of the effectiveness of telemetric interventions in T1DM management and provided insights into the application of telemetric interventions. The evidence for the effectiveness of telemetric approaches in the management of T1DM might be inconsistent. Further studies with a clear and homogeneous methodology are necessary for research and for patients. In addition, we need further research to understand how, why, and when technology can improve the outcomes. Studies should not only focus on HbA_1c_ but also address other outcomes, in particular, fasting blood glucose, blood pressure, QoL, cost-effectiveness, and time saved. Additionally, future studies should provide sufficient statistical power. Further research regarding T1DM is required to examine the special needs of this subgroup in more detail and to develop and adapt suitable interventions. The alarming number of findings with nonsignificant *P* values reveals a need for better study planning as well as RCTs with large sample sizes. In conclusion, telemetry might be a promising approach for people diagnosed with T1DM, especially asynchronous interventions, but its potential should be explored further.

## Appendix

Multimedia Appendix 1 Search terms for the databases.

Multimedia Appendix 2 Detailed summary of each publication selected for inclusion in the systematic meta-review, including all measured outcomes (n=17).

Multimedia Appendix 3 Detailed presentation of all intervention effects (significant and nonsignificant) on the key outcomes.

Multimedia Appendix 4 Quality assessment using A MeaSurement Tool to Assess systematic Reviews (AMSTAR 2) (n=5 studies).

Multimedia Appendix 5 Funnel plot assessing publication bias using HbA1c levels (%) at the end of the study.

## Abbreviations

AMSTAR 2: A MeaSurement Tool to Assess systematic Reviews
DM: diabetes mellitus
DRQoL: diabetes-related quality of life
EPHPP: Effective Public Health Practice Project
HbA_1c_: hemoglobin A_1c_
HRQoL: health-related quality of life
NICE: National Institute for Health and Care Excellence
PRISMA: Preferred Reporting Items for Systematic Reviews and Meta-Analyses
QoL: quality of life
RCT: randomized controlled trial
T1DM: type 1 diabetes mellitus
T2DM: type 2 diabetes mellitus
