# Supplementary material for: Telemetric Interventions Offer New Opportunities for Managing Type 1 Diabetes Mellitus: Systematic Meta-review
Source: JMIR Diabetes. 2021 Mar 16;6(1):e20270. doi: 10.2196/20270 (PMC8080418; doi:10.2196/20270)
Supplement: Multimedia Appendix 1 [file diabetes_v6i1e20270_app1.pdf]

Search terms for the databases.

PUBMED

Keywords	diabetes mellitus, gestational diabetes, telemetry, telemedicine, telemonitoring
Filters	clinical trial, meta-analysis, randomized controlled trial, systematic review; publication date from 2008/01/01 to 2020/12/31; English; German
Search	((((("diabetes mellitus"[Title/Abstract]) OR "Diabetes Mellitus"[MeSH Terms]) OR "gestational diabetes"[Title/Abstract]) OR diabetes, gestational[MeSH Terms])) AND (((((("Telemetry"[Mesh]) OR "Telemedicine"[Mesh]) OR telemonitoring[Title/Abstract]) OR "telemetry"[Title/Abstract]) OR "telemedicine"[Title/Abstract]))

EMBASE

Keywords	diabetes mellitus, pregnancy diabetes mellitus, gestational diabetes, telemetry, telemedicine, telemonitoring
Filters	clinical trial, meta-analysis, randomized controlled trial, systematic review; 2008-2020; English; German
Search	((‘diabetes mellitus’/exp OR ‘pregnancy diabetes mellitus’/exp OR ‘diabetes mellitus’:ab,ti OR ‘gestational diabetes’: ab,ti) AND (‘telemetry’/exp OR ‘telemedicine’/exp OR ‘telemonitoring/exp OR ‘telemedicine:ab,ti OR ‘telemonitoring:ab,ti OR ‘telemetry’:ab,ti))

COCHRANE

Keywords	diabetes mellitus, gestational diabetes, telemetry, telemedicine, telemonitoring
Filters	cochrane reviews, trials and clinical answers; from January 2008 to April 2020; English; German
Search	((MeSH [Diabetes Mellitus] OR MeSH [Diabetes, Gestational] OR “diabetes mellitus”:ti,ab OR “gestatinal diabetes”:ti,ab) AND (MeSH [Telemedicine] OR MeSH [Telemetry] OR “telemedicine”:ti,ab OR “telemetry”:ti,ab OR “telemonitoring”:ti,ab))

CINAHL

Keywords	diabetes mellitus, gestational diabetes, telemetry, telemedicine, telemonitoring
Filters	clinical trial, meta-analysis, randomized controlled trial, systematic review; 2008/01/01-2020/04/02; English, German
Search	((MH “diabetes mellitus” OR TI “diabetes mellitus” OR AB “diabetes mellitus” OR TI “gestational diabetes” OR AB “gestational diabetes”) AND (MH telemedicine OR MH telemetry OR TI telemetry OR AB telemetry OR TI telemedicine OR AB telemedicine OR TI telemoritoring OR AB telemonitoring))

WEB OF SCIENCE CORE COLLECTION

Keywords	diabetes mellitus, gestational diabetes, telemetry, telemedicine, telemonitoring
Filters	2008-2020; English, German
Search	((TOPIC “diabetes mellitus” OR TOPIC “gestational diabetes”) AND (TOPIC telemetry OR TOPIC telemedicine OR TOPIC telemonitoring))
