# Supplementary material for: Telemetric Interventions Offer New Opportunities for Managing Type 1 Diabetes Mellitus: Systematic Meta-review
Source: JMIR Diabetes. 2021 Mar 16;6(1):e20270. doi: 10.2196/20270 (PMC8080418; doi:10.2196/20270)
Supplement: Multimedia Appendix 2 [file diabetes_v6i1e20270_app2.pdf]

Detailed summary of each publication selected for inclusion in the systematic meta-review, including all measured outcomes (n=17).

Author, Year/ Country	Topic	Design	Participants or included studies	Intervention(s)/ (Control)	Duration (Months)	Outcomes/Results	Overall value of intervention(s)
**Systematic reviews and meta-analysis (n=5)**							
(Lee et al. 2017)<br><br>Europe, North America	Telemedicine for the management of glycemic control and clinical outcomes	SR & MA of RCTs	RCTs (n=38)	Telemedicine focus (monitoring, education, consultation, case-management, and peer mentoring)<br><br>Methods for providing feedback included text messages, phone calls, standardized messages, web–based educational modules, SMS, teleconferences<br><br>Studies primarily used telephone, mobile phones, modem, or internet to transmit their data	-	• Positive effects on glycemic control noted with studies examining telemedicine, with a mean reduction of 0.18% (95% CI: 0.04, 0.33; *P*=.01) at the end of intervention<br>• Studies with longer duration (>6 months) who recruited patients with a higher baseline HbA_{1c} (≥9%) were associated with larger effects<br>• Telemedicine interventions that involve individualized assessments, audit with feedback and skill building were also more effective in improving glycemic control<br>• No benefits observed on BP, lipids, body weight, diabetes-related, and health-related QoL	Insufficient:<br><br>For glycemic control and other clinically relevant outcome among T1DM patients
(Viana et al. 2016)<br><br>-	Interventions to improve patients' compliance with therapies aimed at lowering HbA_{1c} in T1DM: clinical trials of psychological, telecare, and educational interventions	SR & MA of RCTs	Telecare RCTs (n=6); Patients (n=494)	Telecare interventions	-	• No decrease in HbA_{1c} after telecare (MD −0.124%; 95% CI, −0.268, 0.020; *P*=.090)	None
(Shulman et al. 2010)<br><br>-	Impact of telemedicine interventions involving routine transmission of blood glucose data with clinician feedback on metabolic control	SR & MA of randomized trials	n=10	Data transmission: Modem, fax, short message service<br><br>Feedback: Phone calls, e-mail, videoconference, shot message service	-	• No significant effect on HbA_{1c} (MD −0.12, 95% CI, −0.35, 0.11), severe hypoglycemia (OR 1.42, 95% CI, 0.22, 9.32), or diabetic ketoacidosis (OR 1.02, 95% CI, 0.24, 4.23)<br>• The limited data available on patient satisfaction, diabetes-related QoL, and cost also suggested no differences between groups<br>• Telemedicine group did not attend the three month visit with a cost savings of $142.00<br>• Indirect costs were considered but not converted into dollar value	None:<br><br>It remains possible that there are other benefits of telemedicine not adequately reported

Author, Year/ Country	Topic	Design	Participants or included studies	Intervention(s)/ (Control)	Duration (Months)	Outcomes/Results	Overall value of intervention(s)
(Edwards et al. 2014)<br><br>US	Interventions, barriers and facilitators to achieving optimal self-care by children with T1DM in educational settings	SR	Telemedicine (n=1)	*See Izquiero 2009*	-	• Exchangig measurements between school nurse with diabetes center nurse via telemedicine was effective in significantly improving diabetes QOL (treatment barriers and treatment adherence) and HbA_{1c}<br>• Telemedicine between health care providers and schools, and individually-tailored support for school children is effective in specific contexts<br>• Telemedicine in school was effective for individual case management	Mildly positive
(Peterson 2014)<br><br>-	Improving type 1 diabetes management with mobile tools	SR	n=14	Interventions: internet–based; mobile–based; telephone service, videoconference	-	• 7 of the 14 articles reported statistically significant decreases in measured outcomes<br>• 11 of the 14 authors (79%) reported success with their intervention<br>• 12 studies reported decrease in HbA_{1c} values in their intervention groups	Positive
**“Real-time video interventions” (n=3)**							
(Freeman et al. 2013)<br><br>-	Behavioral health care for adolescents with poorly controlled diabetes via skype	RCT	IG (n=47)<br>CG (n=45)	IG: internet–based videoconferencing (Skype)<br><br>CG: traditional clinic visits<br>Both groups: 10 sessions lasting 1h to 1.5h	3	• No significant differences in working alliance inventory scores (36-item measure of therapeutic alliance) were found for those receiving behavioral health care via Skype versus in-clinic<br>• Youth working alliance inventory goal and total scores were significantly associated with the number of sessions completed for those in the clinic group	None:<br><br>Behavioral health can be delivered to youth with T1DM via internet–based videoconferencing without significantly impacting the therapeutic relationship
(Marker et al. 2020)<br><br>US	Intervention to reduce hypoglycemia fear in parents of young kids with T1DM through video–based telemedicine	RCT	IG (n=22)<br>CG (n=21)	IG: REDCHiP (Reducing Emotional Distress for Childhood Hypoglycemia in Parents) includes 10 sessions delivered remotely through telemedicine (real-time secure videoconferencing); 7 weekly group sessions and 3 individual sessions; parents participated through personal computer, smartphone, or tablet<br><br>Both: 3 home visits; monthly data transmission	7	• Intervention completers reported high satisfaction with the treatment groups (89% average satisfaction rating)<br>• Parent-reported positive influencers of the REDCHiP intervention were increased knowledge, fear awareness, coping strategies, confidence, behavioral parenting strategies, and support	Positive:<br><br>REDCHiP demonstrated initial feasibility and acceptability
(Bakhach et al. 2019)<br><br>US	Home telemedicine to improve psychosocial outcomes in young adults with diabetes	Prospective cohort pilot study	IG (n=42)<br>CG (n=39)	IG: home telemedicine clinic model (CoYoT1 Clinic = Colorado Young Adults with T1D Clinic, pronounced “coyote”) visits consited of individual appointment with a provider and a group appointment with other young adults using home telemedicine; videoconferencing, internet–based; 3 telemedicine visits and 1 in-person appointment<br><br>CG: individual clinic visits	12	Psychosocial and behavioral outcomes:<br>• CoYoT1 participants reported lower levels of distress (*P*=.03), increased diabetes self-efficacy (*P*=.01), and improved ability to communicate with others about diabetes (*P*=.04) over the study period compared to controls<br>• Males in the control group reported increases in depressive symptoms (*P*=.03) during the study period, but CoYoT1 participants showed no changes	Positive:<br><br>Group home telemedicine positively affects diabetes distress, self-efficacy, and diabetesspecific communication

Author, Year/ Country	Topic	Design	Participants or included studies	Intervention(s)/ (Control)	Duration (Months)	Outcomes/Results	Overall value of intervention(s)
**"Asynchronous interventions" (n=4)**							
(Boogerd et al. 2017)<br><br>Netherlands	Sugarsquare, a web–based patient portal for parents of a child with T1DM	RCT	IG (n=54)<br>CG (n=51)	IG: web–based patient portal, called Sugarsquare; online parent-professional communication, peer support, and disease information; nurse practitioners of the diabetes care team moderate the forum daily, organize weekly chat sessions<br><br>CG: clinic visits 4 times a year	6	• High practicability and integration in all users, moderate acceptability and demand in parents, and high acceptability and demand in health care professionals<br>• Baseline parenting stress index scores were related to the parents' frequency of logging on (*P*=.03) and page-views (*P*=.01)<br>• No significant differences in change in parenting stress between groups (*P*=.49)<br>• No significant differences in change over time in HbA_{1c} levels between groups ($F_{3,101}$=.040, *P*=.84)	Mildly positive:<br><br>Implementing Sugarsquare was partly feasible, given moderate demand and acceptability in parent users and lack of potential efficacy
(Ruiz de Adana et al. 2020)<br><br>Spain	Impact of telemedicine care in patients with T1DM with multiple doses of insulin and suboptimal HbA_{1c}	RCT	IG (n=163)<br>CG (n=167)	IG: 2 face-to-face visits + 1 telemedicine visit using "Diabetic" (= internet–based telemedicine system); data transmission internet–based; feedback via e-mail and mobile phone messages<br><br>CG: 3 face-to-face visits	6	• At month 6, mean change in HbA_{1c} levels was -0.04% ± 0.5% (-0.5 mmol/mol ± 5.8 mmol/mol) in the CG and 0.01% ± 0.6% (0.1 mmol/mol ±6.0 mmol/mol) in the IG (*P*=.4941)<br>• Number of patients who achieved HbA_{1c} <7% (<53 mmol/mol) was 73 and 78 in the CG and IG, respectively; significant differences were not found regarding safety end points at 6 months<br>• Changes in HRQoL between the first visit and final visit did not differ between cohorts, and, regarding fear of hypoglycemia statistically significant differences observed at baseline remained unchanged at 6 months (*P*<.05)	None:<br><br>Telemedicine provides similar efficacy and safety outcomes as face-to-face visits
(Martinez-Sarriegui et al. 2011)<br><br>Spain	How continuous monitoring changes the interaction of patients with a mobile telemedicine system	RCT, cross-over	Patients (n=10)	IG: DIABTel telemedicine system AND real-time Continuous Glucose Monitoring (CGM) was used for 3 days every week during the intervention phase<br>DIABTel: *P*C–based application used by physicians and nurses at the hospital, and palmtop computer (personal assistant) used by patients; feedback within 24 hours<br><br>CG: Patients used DIABTel system	1 each phase (intervention and control) with 1,5 months wash-out period	• Number of sessions established with personal assistant was considerably higher during the intervention period than control period (29.0 versus 18.8, *P*<.05), and it was also higher than the number of Web sessions (29.0 versus 22.2, p < .01)<br>• Number of daily boluses was higher during intervention period than in control period (5.27 versus 4.40, *P*<.01)<br>• Number of daily blood glucose measurements was higher during the intervention period (4.68 versus 4.05, p < .05)<br>• Patients would recommend the use of DIABTel in routine care	Positive:<br><br>use of a continuous glucose monitor changes the way patients manage their diabetes, as observed in increased number of daily insulin bolus, increased number of daily blood glucose measurements

Author, Year/ Country	Topic	Design	Participants or included studies	Intervention(s)/ (Control)	Duration (Months)	Outcomes/Results	Overall value of intervention(s)
(Villarreal Pena et al. 2013)<br><br>Germany	Impact of telemedicine assessment on glycemic variability in children with T1DM	Cohort study	Children (n=13)	3-month telemedical period (telemedicine assessment was performed every 2 weeks) Compared with a subsequent 4-month period without telemedical support (data upload but no support)<br><br>Accu-Chek Smart Pix software; data transmission & feedback via web + SMS	7	• At the end of the assessment phase, mean HbA_{1c} levels were significantly reduced (*P*=.012) with no significant reductions in the Low Blood Glucose Index (*P*=.115) and Average Daily Risk Range (*P*=.552)<br>• No significant increases observed in Mean Blood Glucose (*P*=.861) or the High Blood Glucose Index (*P*=.807)	Positive:<br><br>Telemedical assessment for 3 months in children improved metabolic control, by reducing HbAlc values and, to a lesser extent, by decreasing glycemic variability, without increasing acute complications
**"Combined interventions" (real-time and time shifted communication) (n=4)**							
(Bertuzzi et al. 2018)<br><br>Italy	Teleconsultation in T1DM (TELEDIABE)	RCT	IG (n=37)<br>CG (n=40)	IG: web–based data transmission; teleconsultation with diabetologist via videocommunication; tele-education tools<br><br>CG: standard consultation; not adequately described	12	• Patients did not show a statistical change in HbA_{1c} from baseline at 12th month in both groups (*P*=.56 for CG; *P*=.45 for IG) and between (*P*=.60) groups<br>• IG reduced severe hypoglycemic episodes (*P*=.03) & were largely satisfied with the activities, perceived a good improvement in the self-management, and reported to have time saving (mean time saved for patient 115 min ± 86 min for each visit) and cost reduction (average cost of 80 € for each visit)	Positive
(Laptev und Peterkova 2017)<br><br>Russia	Use of telemedicine improves glycemic control and QoL in T1DM children on insulin pump therapy	RCT	IG (n=20)<br>CG (n=20)	IG: data transmission twice every month via internet; specialist provided recommendations via e-mail, telephone or video call<br><br>CG: usual care<br><br>Both: 3 clinic visits	6	• At 24 weeks, the baseline mean HbA_{1c} (8.7% in the two study groups) had decreased to 7.7% in the IG, as compared with 8.4% in the CG (*P*<.05)<br>• The proportion of patients who reached the HbA_{1c} target (<7,5%) was greater in the IG (50%) than in the CG (20%, *P*<.05)<br>• A number of DRQoL indicators for both parents and children with T1DM at the end of the study compared to baseline significantly increased in the IG compared with the CG (*P*<.05)	Positive:<br><br>In children with inadequately controlled T1DM, telemedical support proved to be feasible and resulted in significant improvement in glucose control (HbA_{1c}, glucose variability) and QoL
(Yaron et al. 2019)<br><br>Israel	Comparing a telemedicine therapeutic intervention with routine care in adults with T1DM mellitus treated by insulin pumps	RCT	IG (n=37)<br>CG (n=37)	IG: data transmission monthly via Carelink Pro Software (internet–based); immediate phone feedback + face-to-face visits one in 6 months<br><br>CG: face-to-face visits once every 3 months	12	• Mean changes in HbA_{1c} adjusted to baseline – 0.08% (0.25 mmol/mol) vs. – 0.01% (0.03 mmol/mol), in IG and CG, respectively (*P*=.18) at 12 months, without an increased frequency of hypoglycemie<br>• IG felt satisfied and interested in continuing with treatment (*P*=.04)<br>• Direct total costs 24% less in IG, and indirect total costs decreased by 22% compared to the year preceding the study	Neutral:<br><br>internet–based insulin dose adjustment is as effective and safe as routine care

Author, Year/ Country	Topic	Design	Participants or included studies	Intervention(s)/ (Control)	Duration (Months)	Outcomes/Results	Overall value of intervention(s)
(Gandrud L. et al. 2018)<br><br>US	Intensive remote monitoring versus conventional care	RCT	IG (n=60)<br>CG (n=57)	IG: Intensive remote monitoring (IRT); data transmission via web/app/device/phone; IRT consisted of weekly remote review of uploaded data by research staff (diabetes educator, nurse practitioner, and/or physician); feedback via e-mail and/or telephone<br><br>Both: regular quarterly clinic visits, uploading device data to the clinic on a weekly basis, and receiving automated weekly text reminders	6	• Mean (SD) 6-month HbA_{1c} change for IRT vs CC was −0.34% (0.85) (−3.7 mmol/mol) vs −0.05% (0.74) (−0.5 mmol/mol) overall (*P*=.071)<br>• −0.15% (0.67) (1.6 mmol/mol) vs −0.02% (0.66) (0.2 mmol/mol) for ages 8 to 12 (*P*=.541)<br>• and −0.50% (0.95) (−5.5 mmol/mol) vs −0.06% (0.80) (−0.7 mmol/mol) for ages 13 to 17 (*P*=.056)<br>• Diabetes-related QoL increased by 6.5 points and 1.3 points for IRT and CC, (*P*=.062)<br>• 3 months after intervention, HbA_{1c} changed minimally among treated children aged 8 to 12 but increased by 0.22% (0.89) (2.4 mmol/mol) among those aged 13 to 17	Positive:<br><br>IRT substantially affected diabetes metrics and improved QoL among pediatric patients with T1DM Adolescents experienced a stronger treatment effect, but had difficulty in sustaining improved control after intervention cessation
**Not specified (n=1)**							
(Chorianopoulou et al. 2015)<br><br>Greece	Investigation of the quality and effectiveness of telemedicine in children	Qualitative	IG (n=50 parents)<br>CG (n=50 parents)	Intervention/telemedicine system (home telemonitoring) not adequately described (telemedicine and online education)	-	• Majority of parents thinks their knowledge level on telemedicine system is sufficient (96%) and would recommend its use (82%)<br>• 80% evaluate the telemedicine system as adequate	Positive:<br><br>Study demonstrated improved access to specialized health care via telemedicine in combination with online education improved health status and reduced hospitalizations and emergency department visits

Notes:

Green = telemedicine additional to usual care

Abbreviations:

BP = blood pressure; CG = control group; CoYoT1 Clinic = colorado young adults with T1DM clinic; DRQoL = diabetes-related quality of life; eHealth = electronic Health; FBG = fasting blood glucose; HbA_{1c} = hemoglobin A1c; HRQoL = health-related quality of life; IG = intervention group; IRT = intensive remote monitoring; MA = meta-analysis; MD = mean deviation; mHealth = mobile Health; N/S = Not significant; OR = odds ratio; QoL = quality of life; RCT = randomized controlled trial; REDCHiP = reducing emotional distress for childhood hypoglycemia in parents; SD = standard deviation; SMBG = self monitoring blood glucose; SMS = short message service; SR = systematic review; T1DM = type 1 diabetes mellitus.
