# Supplementary material for: Telemetric Interventions Offer New Opportunities for Managing Type 1 Diabetes Mellitus: Systematic Meta-review
Source: JMIR Diabetes. 2021 Mar 16;6(1):e20270. doi: 10.2196/20270 (PMC8080418; doi:10.2196/20270)
Supplement: Multimedia Appendix 3 [file diabetes_v6i1e20270_app3.pdf]

Detailed presentation of all intervention effects (significant and nonsignificant) on the key outcomes.

Diabetes type	Intervention	Design	Reference	HbA_{1c}	BP	Body weight	DR QoL	HR QoL	Cost-effective ness	Time saving	Other
**T1DM (n=17)**	**Not categorized**	**SR & MA**	(Lee et al. 2017)	+++	000	000	000	000			
			(Viana et al. 2016)	000							
			(Shulman et al. 2010)	000			000		000		
			(Edwards et al. 2014)	+++			+++				
			(Peterson 2014)	+++							
	**"Real-time video communication"**	**RCT**	(Freeman et al. 2013)								x
			(Marker et al. 2020)								x
		**Cohort, pilot**	(Bakhach et al. 2019)								x
	**"Asynchronous communication"**	**RCT**	(Boogerd et al. 2017)	(++)							
			(Ruiz de Adana et al. 2020)	(00)				(00)			
			(Martinez-Sarriegui et al. 2011)								x
		**Cohort**	(Villarreal Pena et al. 2013)	+							
	**"Combined forms of communication" (asynchronous and real-time)**	**RCT**	(Bertuzzi et al. 2018)	(0); (00)					+ (S N/R)	+ (S N/R)	
			(Laptev/Peterkova 2017)	++			++				
			(Yaron et al. 2019)	(++)					++ (S N/R)		
			(Gandrud L.M. et al. 2018)	(++)			(++)				
	**Not specified**	**Qualitative**	(Chorianopoulou et al. 2015)								x

Notes:

x = study examined other than the selected outcomes
+++ = overall positive effects (SR/ MA)
++ = improvement in intervention group compared to control group (intergroup)
+ = improvement in intervention group compared to baseline (intragroup)

1 = inconclusive
000 = no overall effect (SR/MA)
00 = no changes in intervention group compared to control group (intergroup)
0 = no changes in intervention group compared to baseline (intragroup)
- - = deterioration in intervention group compared to control group (intergroup)
- = deterioration in intervention group compared to baseline (intragroup)
( ) = not statistically significant
Yellow = intervention additional to usual care
Green = all groups with telemedical support

Abbreviations:

BP = blood pressure; d = diastolic blood pressure; DL = diabetes-related; HbA_{1c} = hemoglobin A_{1c}; HL = health-related; MA = meta-analysis; QoL = quality of life; RCT = randomized controlled trial; s = systolic blood pressure; S N/R = significance not reported; SR = systematic review; T1DM = type 1 diabetes mellitus.
