# Supplementary material for: Telemetric Interventions Offer New Opportunities for Managing Type 1 Diabetes Mellitus: Systematic Meta-review
Source: JMIR Diabetes. 2021 Mar 16;6(1):e20270. doi: 10.2196/20270 (PMC8080418; doi:10.2196/20270)
Supplement: Multimedia Appendix 4 [file diabetes_v6i1e20270_app4.pdf]

Quality assessment using "A MeaSurement Tool to Assess systematic Reviews" (AMSTAR 2) (n=5 studies)

**Question/ Study**	PICO	Priori Design	Selec-tion study designs	Compre-hensive search strategy	Study selec-tion in dupli-cate	Data extrac-tion in dupli-cate	List of exclu-ded studies	Des-cription of studies	Assessm ent of risk of bias	Sorces of fun-ding	Statis-tical combi nation	Impact of risk of bias on results	Account for risk of bias when inter-preting results	Hetero-geneity	Publi-cation bias	Conflict of interest	**Overall assess-ment**
**Lee et al. 2017**	+	+	+	o	+	+	o	+	+	-	+	+	+	+	+	+	**High**
**Shulman et al. 2010**	+	+	+	o	+	+	+	+	+	-	+	+	+	+	+	+	**High**
**Viana et al. 2016**	-	+	-	o	+	+	-	+	+	-	+	+	+	+	+	+	**Moderate**
**Edwards et al. 2014**	+	+	+	o	+	-	-	+	+	-	/	/	+	+	/	+	**Moderate**
**Peterson 2014**	+	+	+	o	-	-	+	+	-	-	/	/	-	+	/	+	**Critically low**

+ = criterion met, - = criterion not met, o = criterion partially met, / = not applicable

Quality assessment using "Effective Public Health Practice Project" (EPHPP) (n=17 studies)

Question/ Study	A Seletion Bias (Q1)	A Selection Bias (Q2)	A SCORE	B Study Design	B SCORE	C Confoun-ders (Q1)	C Confoun-ders (Q2)	C SCORE	D Blinding (Q1)	D Blinding (Q2)	D SCORE	E Data collection (Q1)	E Data Collection (Q2)	E SCORE	F Withdrawals and Drop-outs (Q1)	F Withdrawals and Drop-outs (Q2)	F SCORE	GLOBAL RATING
**"Real-time video interventions"**																		
**Freeman et al. 2013**	4	2	**	1	***	2	/	***	3	3	**	1	1	***	1	2	**	**High**
**Marker et al. 2020**	1	5	**	1	***	2	/	***	3	3	**	1	1	***	1	1	***	**High**
**Bakhach et al. 2019**	1	5	**	3	**	2	/	***	3	3	**	1	1	***	1	2	**	**High**
**„Asynchronous interventions"**																		
**Boogerd et al. 2017**	4	3	*	1	***	2	/	***	3	3	**	1	1	***	1	2	**	**Moderate**
**Ruiz de Adana et al. 2020**	4	5	*	1	***	2	/	***	3	3	**	1	1	***	1	1	***	**Moderate**
**Villarreal Pena et al. 2013**	4	5	*	5	**	2	/	***	3	3	**	1	1	***	1	1	***	**Moderate**
**Martinez-Sarriegui et al. 2011**	3	5	*	1	***	2	/	***	3	3	**	1	1	***	2	1	***	**Moderate**
**„Combined intervention"**																		
**Yaron et al. 2019**	4	1	**	1	***	2	/	***	3	3	**	1	1	***	1	1	***	**High**
**Bertuzzi et al. 2018**	4	5	*	1	***	2	/	***	3	3	**	3	3	**	1	1	***	**Moderate**
**Laptev and Peterkova 2017**	4	5	*	1	***	2	/	***	3	3	**	1	1	***	1	1	***	**Moderate**
**Gandrud et al. 2018**	4	5	*	1	***	2	/	***	3	3	**	1	1	***	1	4	*	**Weak**

* = weak, ** = moderate, *** = strong, 1-5 = response options according to EPHPP, / = not applicable

Quality assessment using "National Institute for Health and Care Excellence (NICE) for qualitative studies" (n=1 study)

**Question/ Study**	Is a qualitative approach appropriate?	Is the study clear in what it seeks to do?	How defensible/ rigorous is the research design/ methodology?	How well was the data collectio carried out?	Is the role of the researcher adequately described?	Is the context clearly described?	Were the methods reliable?	Is the data analysis suffi-ciently rigorous?	Is the data "rich"?	Is the analysis reliable?	Are the findings convincing?	Are the finding relevant to the aims of the study?	Con-clusions	How clear and coherent is the re-porting of ethics?	**Overall assess-ment**
**Choriano-poulou et al. 2015**	Appropriate	Clear	Defensible	Appropri ately	Not decribed	Unclear	Reliable	Rigorous	Poor	Not reported	Convincing	Relevant	Adequate	Not reported	**+** **(Moderate)**
