# Supplementary material for: Telemetric Interventions Offer New Opportunities for Managing Type 1 Diabetes Mellitus: Systematic Meta-review
Source: JMIR Diabetes. 2021 Mar 16;6(1):e20270. doi: 10.2196/20270 (PMC8080418; doi:10.2196/20270)
Supplement: Multimedia Appendix 5 [file diabetes_v6i1e20270_app5.pdf]

Funnel plot assessing publication bias using HbA1c levels (%) at the end of the study (n=6 RCTs)

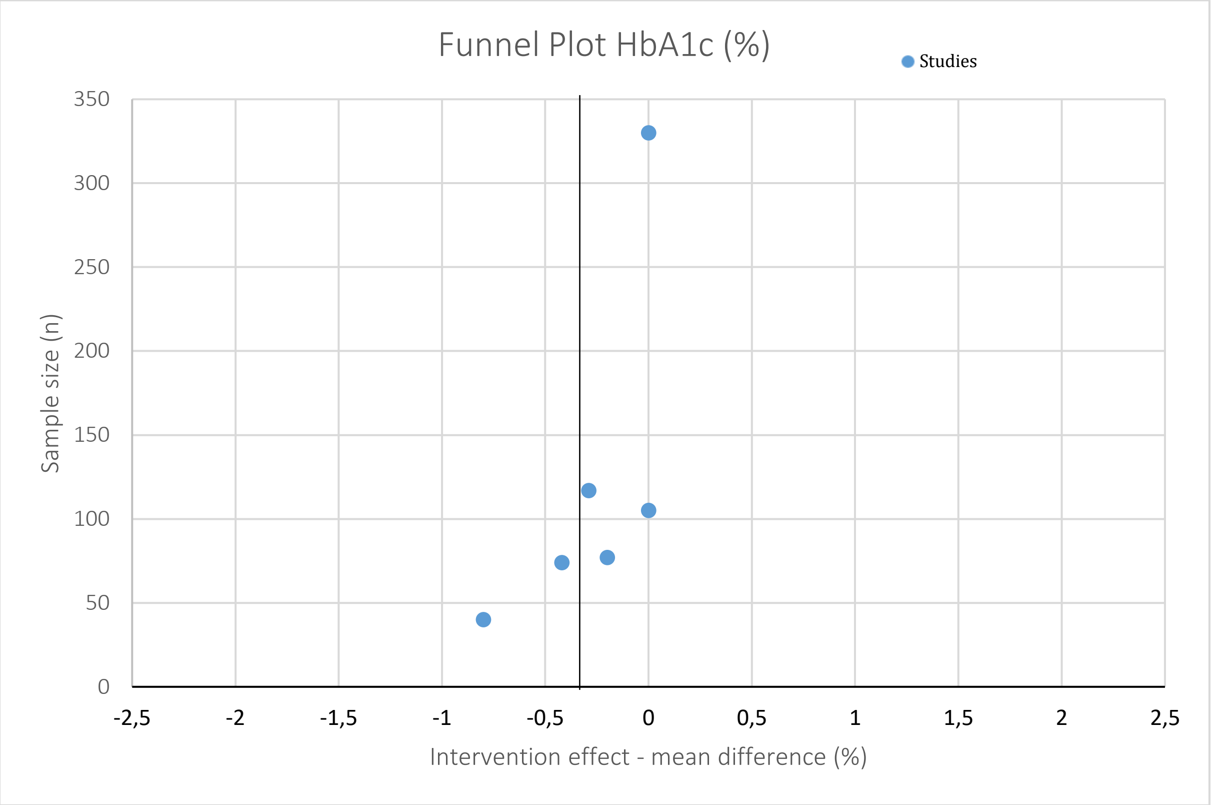

Study	Sample size, n	Intervention group (mean), %	Control group (mean), %	Mean Difference (MD), %
Boogerd et al. 2017	105	7,9	7,9	0
Ruiz de Adana et al. 2020	330	7	7	0
Bertuzzi et al. 2008	77	7,3	7,5	-0,2
Laptev/Peterkova 2017	40	7,7	8,5	-0,8
Yaron et al. 2019	74	7,5	7,92	-0,42
Gandrud et al. 2018	117	8,56	8,85	-0,29
